# Macon Medical Society

**Published:** 1885-03

**Authors:** 


### Society Reports.

---

### MACON MEDICAL SOCIETY.

Reported for The Atlanta Medical and Surgical Journal.

Stated Meeting, December 16, 1884.

Dr. J. P. Stevens in the chair.

Dr. Keenan Hall opened the discussion by reading the following paper on

### MASTITIS.

The mammary glands in the woman are situated on either side of the thorax and in front, intimately connected with the pectoralis major muscles. In the language of Dr. Meigs, "the mammary gland is suspended by the skin in front of the thorax." It is circular in form, projecting, as a rule, three or four inches, according to the amount of adipose tissue. It is broader at the base and gradually tapers to a point. It is composed of gland structure and is divided into lobes, these are further divided into lobules, held together by fibrous connective tissue. From these lobes ducts are sent out which, uniting, form larger ducts ; these still further unite till finally they terminate at the apex or nipple into four or five mouths.

The gland is intended to secrete the milk. It can be detected at an early period of fœtal existence. From birth till about the 13th year they remain undeveloped, being about as large in one sex as in the other. At this age with the development of the generative organs they, in the female, become more and more prominent till the 16th year, when they have nearly attained their full size, though they are never completely developed till 20. During gestation they become active and contain fluid. They remain active till after the period of suckling, when they gradually diminish, though they never return to the virgin size. After the child is born and put to the breast the irritation causes more blood to be sent to the part and at the same time a change takes place in this watery secretion, converting it into milk. The glands become swollen, tense and painful, which the act of nursing increases. The woman does not allow the child to fully empty the breast. Gradually the liquid portion is pressed out, leaving the solid residue, which are familiarly known as milk-clots; they irritate and inflame the breast, and if not attended to in time will result in an abscess.

The causes of mammitis, according to the latest authorities, are: Lactation, epidemic influence, exposure to cold, inflammation of the nipple extending to breast, repression of the secretion of milk at an early period, obstructed lacteal ducts, mental disturbances, bruises and injuries, and probably irregularities of diet.

Dr. Fordyce Barker gives three varieties of mammary abscess according to its anatomical seat. It may be (1) in the subcutaneous areolar tissue; (2) in the gland itself; (3) in the areolar tissue between the gland and the thoracic wall.

The prognosis is as a rule favorable, though failure may attend the most skillful treatment.

As in all inflammatory affections, mammary adinitis may be ushered in with chill, fever and all the other symptoms attendant upon an inflammation. Various remedies have been employed from time to time for the cure of this affection, viz: Belladonna, bryonia, conium, phytolacca, iodide potassium, quinine, aconite, spirit mindereri, sulphide calcium.

Belladonna will repress the secretion of milk and cause a relaxation of the muscles of the lacteal ducts. It is used externally in the form of plaster, ointment or fluid extract. "Bryonia is indicated where the breasts are swollen, tender, knotty and painful. It will resolve the inflammation and prevent the formation of an abscess." (Hughes.) "Externally it is applied as a rubefacient in glandular engorgements." (Stillé.) It is used both externally and internally. It is spoken of very highly by the homeopaths.

"Conium causes the mammary gland to cease secreting and to become atrophied. D'Outrepont asserts that the breasts will never again secrete milk." (Stillé.)

"Phytolacca is not only useful in simple and in inflammatory engorgements, causing rapid suppuration, but is valuable in those cases where suppuration has already commenced. Here it reduces the inflammation and increases the activity of the absorbents and will often condense an apparently large abscess into the smallest dimensions. It is often the case that neglected or ill-treated mammary abscesses degenerate into ill-conditioned, fistulous ulcers. "I have," says Dr. E. M. Hale, "seen the best results follow the judicious use of this remedy in such cases."

Iodide of potassium, in large and frequently repeated doses, is supposed by some to repress the secretion of milk.

Quinine and aconite are indicated where there is fever.

Sulphide calcium is spoken of very highly by Dr. Ringer, in all inflammatory affections tending to suppuration. Even after the abscess has formed, it has the property of making the pus more laudable. It shortens the duration of the attack, thereby affording a safer convalescence. At first it increases the pain of these affections.

Water dressing, to which may be added alcohol or vinegar, are indicated in local inflammatory congestions.

The congested gland should be supported. This can easily be done by means of one or two adhesive strips about one inch wide and about 16 inches long. In applying them you should raise the breast to its normal position, place one end on the side of the gland, carry around on the other side and as high as the clavicle. Two such straps applied on either side of the mammæ will relieve the pain and will materially aid in our treatment. This of itself will sometimes abort an inflammation. By this means you give the diseased gland as much rest as possible. As soon as the abscess has formed resort should be had to the lancet. If the woman objects to the lancet and the abscess is of the subcutaneous variety you may let the abscess burst of its own accord. Should, however, the abscess be of the glandular or sub-glandular variety, the moment that you are sure pus has formed, you should insist upon the use of the lancet.

In lancing the breast, the antiseptic method of Mr. Lister should be conformed to as much as possible. He lays over the breast, as soon as he withdraws his knife, a piece of cloth previously saturated in carbolated linseed oil, and lets the pus ooze out on the side, where he puts a piece of absorbing material for purposes of cleanliness. In this way air is prevented from entering the cavity ; the possible formation of a series of abscesses is lessened; the woman is saved a tedious convalescence. Of late years Mr. Lister makes a paste of this carbolated oil with carbonate of lime, which he puts on a piece of card-board or tin of whatever size he desires and applies that to the breast as he would the cloth. In the latter stages of mammary adinitis the woman should be placed upon the best tonic treatment, hypophosphites, cod-liver oil, etc.

Dr. Holt : " Abscess of the breast is a very troublesome disease to deal with, and to prevent its developing, a great deal depends upon the early care of the breast after the delivery. Obstruction of the lacteal tubes resulting from sore nipple, is in my mind, the most frequent cause of the difficulty. It is usually preceded by milk fever. Early in the case I try to control the fever by antipyretic doses of quinine and aconite. This I alternate with four to five drop doses of the fluid extract of the phytolacca decandra for its specific action on the inflamed swollen gland. The swollen tender mamma is supported and an ointment of the phytolacca is used on the gland. In my early practice I used as an abortive treatment, muriate of ammonia and vinegar, locally, with very good success. I have never used bryonia. A few years ago my attention was directed to the phytolacca as a remedial agent in this affection. I first used the fluid extract internally in small repeated doses, and then afterwards I had one of our druggists to prepare me an ointment of the powdered root. This I have been using ever since with the most satisfactory results. I have, I believe, with this drug, frequently succeeded in arresting the disease.

When the disease cannot be aborted, and it has gone to suppuration, the lancet should be used ; a valvular opening should be made and the knife passed up to the abscess cavity. Care should be taken not to cut or destroy any lacteal tube, and to avoid going too near the nipple, for the cicatrix will cause retraction and loss of the use of the nipple.

Dr. Fitzgerald : Dr. Hall, in his paper, has covered the whole subject. As to the cause, it is my impression that the trouble arises most frequently from stroking the swollen, tender breast early after confinement by an ignorant nurse. The breast, when full, should be relieved, but this thing of stroking it irritates the gland and does harm. Nor do I allow a breast pump or tube to be used. If the lacteals are obstructed and the breast requires relief, I require the nurse to relieve it by nursing it with her own mouth; this is more in accordance with nature, and we should follow out her indications. Another cause of trouble is, the child is allowed to nurse too frequently. The constant tugging and pulling at the nipple irritates the breast, and in nine out of ten cases causes sore nipple, and an inflamed breast follows. I never allow a young mother to nurse her child more than two or three times daily, and only once or twice during the night, and I insist upon this during the first sixty hours—before the milk is secreted. I have pursued this course for years and I can see its benefits in my practice. I have never seen the quantity of secretion in the breast during the first sixty hours after labor stated, yet I believe it is small in amount, not perhaps over one or two ounces; now, what can be the use of allowing the child to irritate the gland by frequent nursing, when there is so little secretion. In regard to treatment, I will say that I have used phytolacca, but with no benefit whatever. The gland should be well supported and compressed by bandages or otherwise ; the fever should be controlled and pain relieved. If an abscess forms it should be opened at its lowest point for drainage to take place. If it is opened above or too high up there is sure to be trouble. One of the worst cases I ever saw was in a young lady after her first confinement. The cause of the trouble was, I think, kneading and stroking the breast by an ignorant nurse. The abscess formed in the connective tissue under the gland, between it and the chest wall. The abscess finally discharged itself through a sinus between the tubules and was a long time in recovering.

Dr. Moore : Dr. Dugas, of Augusta, proposed to strap the breast, on the theory that a roller-bandage, well applied to a part or organ, relieves pain and prevents or limits inflammatory action. This treatment has been pursued under my own observation with very good results. To prevent or abort mastitis I have often painted the breast with tincture of iodine and applied over it belladonna ointment or plaster ; this has had excellent effect. I have used the belladonna to relieve pain and arrest the secretion of milk, and in two or three cases it has acted well. While the treatment with iodine and belladonna is being employed I allow, under proper precautions, the child to nurse, and if there are reasons to apprehend poisoning, I have the attendant to nurse the breast.

Dr. C. H. Hall : I cannot agree with Dr. Fitzgerald in regard to nursing the child. I believe the child should be allowed to nurse every three or four hours. The child will relieve the breast of the first secretion or colostrum, and the suction of the child's mouth every three hours will sufficiently harden the breast to prevent trouble. A long experience has led me to believe this to be the safest course. I cannot treat a rising breast without phytolacca decandra. I am inclined to think the reason Dr. Fitzgerald has failed to gain success with the drug is, the preparation which he has used is the fluid extract of the old dry plant. The juice of the whole plant is best, and the action in mastitis in properly regulated doses, equals quinine in malarial fever. I prefer Merril's preparation. As to local applications, I have used phytolacca locally during the past two years. I prefer the belladonna plaster for its local action and support of the glands. If I wish to use support alone I employ a broad band. Dr. Fitzgerald is mistaken as to the amount of colostrum secreted. I think it is considerably more than an ounce during the first sixty hours after labor.

After the discussion the secretary read the following report on

MURIATE OF COCAINE.

By Dr. McHatton : I succeeded in procuring a 2 per cent. solution of hydrochlorate of cocaine last week which I have used in the following cases, applying it in each with a camel's hair brush three times with an interval of five minutes between the applications.

1. Case of hypertrophy of the mucosa of the inferior turbinated bane. After the third application the mucosa was so contracted that I could hardly tell where to cauterize. I cauterized the parts thoroughly with actual cautery, and though the patient complained of an unpleasant sensation, he said it did not amount to pain.

2. Also, a case of hypertrophy which was treated with glacial acetic acid without complaint. In this case the application of cocaine was followed by the contraction of the swollen mucosa.

3. Case of specific ulceration of the mouth burnt with the solid stick nitrate of silver. The patient complained of no pain but said he experienced a sensation of warmth and did not taste the caustic for some minutes.

I think that in this agent we have a most valuable local anæsthetic, and thus far we have no idea how universal its use may become.

I wish to call attention particularly to its action on the hypertrophied mucosa of the nose in driving out the blood from the enlarged sinuses, which was first noticed by Dr. Frank Basmorth, of New York city.

Through this action we will have a most valuable agent for the treatment of acute colds, hay fever, and in fact all cases of turgescence of the mucosa of this cavity, which will at least give relief to a most unpleasant symptom.

